# Viscera Preservation in Medical Board Autopsies: Timelines, Outcomes, and Systemic Inefficiencies in Toxicological Analysis

**DOI:** 10.7759/cureus.92886

**Published:** 2025-09-21

**Authors:** Anand Kumar, Ankur Chaudhary, Ajay K Bhagat, Kumar Shubhendu, Sawan Mundri

**Affiliations:** 1 Department of Forensic Medicine and Toxicology, Rajendra Institute of Medical Sciences, Ranchi, IND

**Keywords:** forensic science laboratory, forensic toxicology, medical board autopsy, postmortem analysis, viscera preservation

## Abstract

Background

Viscera preservation for toxicological analysis is a standard practice in medicolegal autopsies, yet its forensic and judicial value amidst delayed evaluations remains contested. This study investigates the impact of such delays on the relevance of toxicological findings, examining the frequency of positive results in relation to report turnaround times. By describing patterns of viscera preservation and toxicological reporting timelines, the research intends to highlight practical challenges and inefficiencies, providing a foundation for evidence-based improvements in forensic protocols.

Methodology

A retrospective observational study was executed, scrutinizing records of medicolegal autopsies performed by medical boards from April 2015 to March 2020. Information regarding the preservation of visceral organs, timeframes for dispatch, and the receipt of toxicological reports was meticulously gathered. Statistical evaluations were conducted to study the correlation between delays in reporting and toxicological outcomes.

Results

Considerable delays were noted in the processes of dispatching preserved viscera and obtaining toxicology reports; a mere 22% of reports were acquired within a six-month period, while 42.5% extended beyond a year. A chi-square statistical analysis indicated a significant relationship (p = 0.039) between extended delays and negative toxicological outcomes. Around 76.9% of the reports yielded negative results, with the presence of ethyl alcohol contaminated by methyl alcohol representing the predominant positive finding (13.5%).

Conclusion

Systemic deficiencies in the preservation of visceral organs and the subsequent toxicological assessment significantly compromise forensic efficacy. A more discerning methodology for preservation, augmented inter-agency collaboration, and innovations in forensic technologies are imperative for enhancing operational efficiency and dependability, along with facilitating prompt and precise toxicological evaluations.

## Introduction

The preservation of viscera for toxicological examination constitutes a pivotal component of medicolegal autopsies, especially in scenarios involving potential poisoning or intoxication, or when the cause of death remains ambiguous. Whenever there is a suspicion of poisoning, the assessment of internal organs can affirm the presence of poisons and support in clarifying the cause of death, which is significant for the resolution of criminal investigations [1,2]. Hence, implementing standardized guidelines for the sourcing, preservation, and scrutiny of specimens is imperative for ensuring the integrity and comparability of toxicological discoveries. Harmonized protocols may effectively mitigate challenges such as delays and resource intensiveness while fostering consistency across various jurisdictions [3].

Medical boards composed of specialists from diverse medical disciplines, presided over by an autopsy surgeon, are constituted for performing medicolegal autopsies, particularly in cases of custodial deaths and other deaths where the implications, both medico-legal and judicial, are significant. Such autopsies effectively integrate the expertise of various trained professionals with the tenets of medical jurisprudence. The implications are considerably heightened in instances where poisoning is suspected in medical board autopsies, as poisoning consistently represents a somewhat ambiguous domain within medical science, especially due to the associated atypical presentations encountered in both clinical and forensic contexts, necessitating verification from Forensic Science Laboratories and entailing preservation of viscera for toxicological analysis.

One of the notable impediments associated with the preservation of viscera is the protracted delay in the acquisition of toxicological results, which obstructs the advancement of legal proceedings and the effective administration of justice [2]. The procedures involved in the collection, preservation, and analysis of viscera are notably resource-intensive. These processes necessitate specialized equipment, adequately trained personnel, and a considerable investment of time. In some cases, alternative specimens such as cerebral tissue or cardiac blood may provide comparable or more timely toxicological findings [4,5].

The significant delays ultimately culminate in a situation where the preponderance of toxicological analysis reports are rendered negatory and devoid of value, notwithstanding the fact that in the majority of such instances, the features of poisoning had already been meticulously observed by autopsy surgeons. This concern received judicial recognition in Criminal Appeal No. 259 of 2009 (Joshinder Yadav vs State of Bihar), where the Honourable Supreme Court of India emphasized the necessity of timely toxicological examination for establishing a definitive cause of death in poisoning cases, noting that procedural delays undermine forensic evidence quality and judicial proceedings [6]. The present study seeks to highlight a long-standing challenge faced by autopsy surgeons: their inability to conclusively opine on the cause of death in poisoning cases by assessing the patterns and timelines of viscera preservation and toxicological reporting in medical board autopsies and evaluating their practical utility in establishing the cause of death.

## Materials and methods

**Figure 1:**
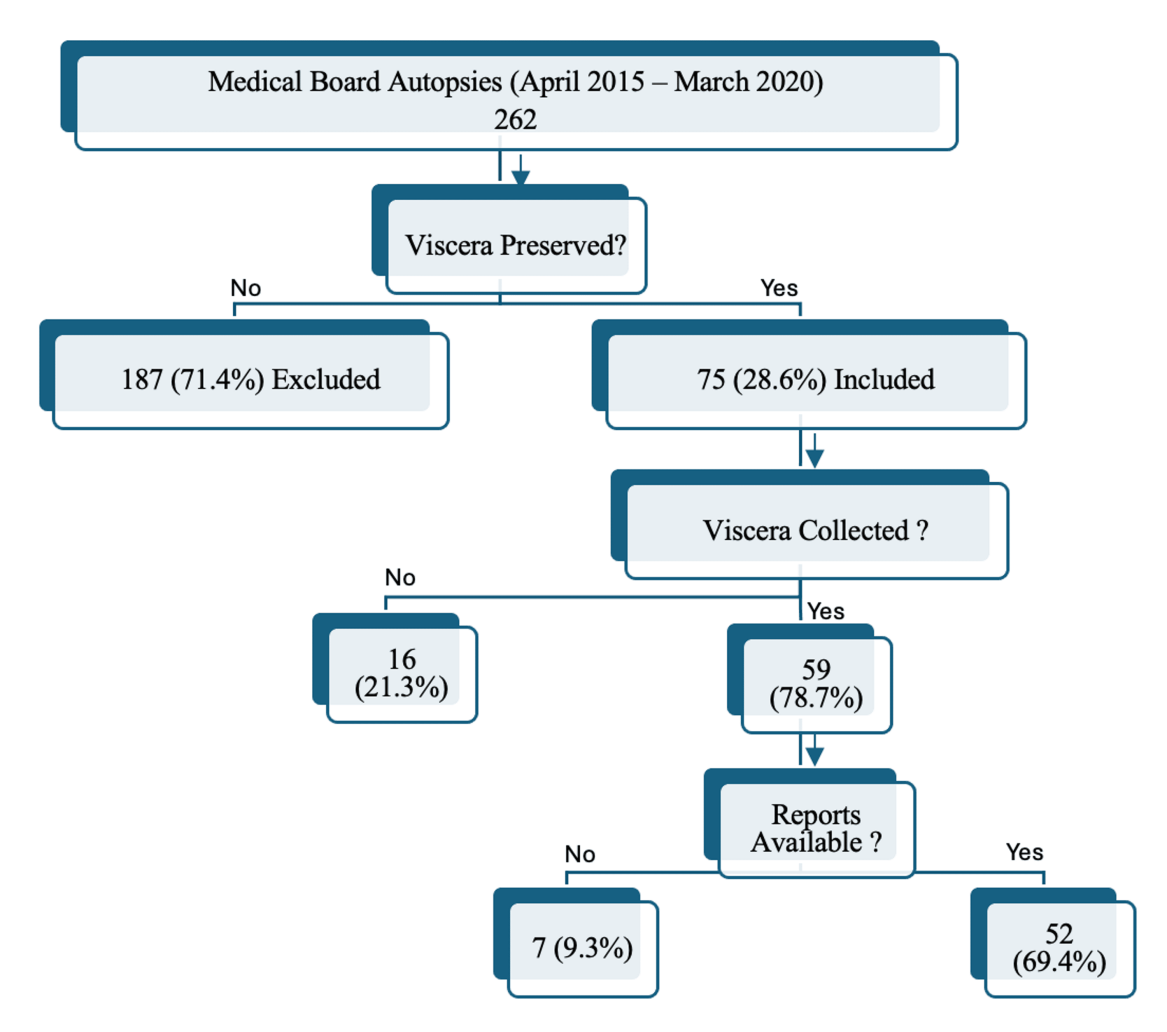
Flow diagram of case selection for analysis of viscera preservation and toxicological reporting in medical board autopsies (April 2015–March 2020).

This retrospective observational study reviewed all medicolegal autopsies conducted by officially constituted medical boards at the Rajendra Institute of Medical Sciences, Ranchi, India, between 1 April 2015 and 31 March 2020. The sampling frame comprised all consecutive medical-board autopsies during this period. Autopsy records were screened to determine whether viscera had been preserved for toxicological analysis. Cases were included if viscera were preserved and the corresponding toxicology reports or laboratory acknowledgements could be traced, and were excluded if viscera were not preserved. The stepwise selection of cases is depicted in Figure 1. Key variables extracted from the records included date of autopsy, date of viscera handover to the investigating officer, date of receipt at the Forensic Science Laboratory (FSL), date of toxicology report issuance, and toxicology result (positive/negative and substance identified).

Two investigators independently abstracted data using a standardized proforma, with discrepancies resolved by consensus after re-review of source documents. Data were entered into Microsoft Excel and analyzed with IBM SPSS Statistics for Windows (Version 26.0; IBM Corp., Armonk, NY). Descriptive statistics were generated, and associations between categorical variables were assessed using the chi-square test with a significance threshold of p < 0.05. No personal identifiers were utilized throughout the stages of data acquisition, documentation, or analysis, thereby guaranteeing the absolute confidentiality of the collected data. Ethical approval for the conduct of this study was duly obtained from the Institutional Ethics Committee (IEC) of the Rajendra Institute of Medical Sciences, Ranchi, Jharkhand.

## Results

A cumulative total of 262 medicolegal autopsies were performed by medical boards over the span of five years. Viscera were preserved in 28.6% (n=75) of the autopsies conducted. During the autopsy reviews, 69.4% (n=52) had accessible viscera reports, while in 9.3% (n=7), these reports were not received, and in 21.3% cases (n=16), the police still needed to acquire the viscera for sending to the Forensic Science Laboratory. Merely 10.7% (n=8) of the preserved viscera were received by the police within one month following the date of the autopsy. Overall, 32% (n=24) of the preserved viscera were obtained by the police in the timeframe of one to three months post-autopsy, with 36% (n=27) being received after three months had passed since the autopsy date (Tables 1-3).

**Table 1:** Distribution of autopsies performed by medical boards according to status of preservation of viscera for toxicological analysis

Viscera preserved	Frequency (n=262)	Percentage (%)
Yes	75	28.6
No	187	71.4
Total	262	100

**Table 2:** Distribution of autopsies performed according to outcome of viscera preserved

Outcome of viscera preserved	Frequency (n=75)	Percentage (%)
Viscera reports available	52	69.4
Viscera reports awaited	7	9.3
Viscera not received by police	16	21.3
Total	75	100

**Table 3:** Distribution of autopsies performed according to duration (from autopsy date) after which viscera were received by Police/concerned authorities

Duration (from autopsy date)	Frequency (n=75)	Percentage (%)
Not yet received	16	21.3
Within 1 month	8	10.7
> 1 – 3 months	24	32.0
> 3 – 6 months	16	21.3
> 6 – 12 months	6	8.0
> 12 months	5	6.7
Total	75	100

As of 31.03.2025, more than five years had transpired (from the date of autopsy) in all 16 autopsies where the viscera had yet to be obtained by the law enforcement authorities for submission to the Forensic Science Laboratory for toxicological examination. Just 22% (n=13) of the viscera analysis reports were received within six months post-autopsy; 23.7% (n=14) trickled in between six and 12 months, while a considerable 42.5% (n=25) arrived after a year. Furthermore, 11.8% (n=7) of the reports had still not been received. More than five years had transpired (from the date of autopsy) as of 31.03.2025 in all seven autopsies, in which the viscera report had not yet been acquired from the Forensic Science Laboratory (Tables 4-6).

**Table 4:** Distribution of autopsies performed according to duration (from autopsy date) after which viscera was not yet received by Police/concerned authorities (until 31.03.2025)

Duration (from autopsy date)	Frequency (n=16)	Percentage (%)
5 – 6 years	5	31.3
> 6 – 7 years	1	6.2
> 7 - 8 years	5	31.3
> 8 – 9 years	4	25
> 9 years	1	6.2
Total	16	100

**Table 5:** Distribution of autopsies performed according to duration (from autopsy date) after which viscera report was received

Duration (from autopsy date)	Frequency (n=59)	Percentage (%)
Within 6 months	13	22
> 6 – 12 months	14	23.7
> 12 - 18 months	4	6.8
> 18 - 24 months	4	6.8
> 24 – 30 months	1	1.7
> 30 – 36 months	3	5.1
> 36 – 42 months	10	17
> 42 – 48 months	2	3.4
> 48 – 54 months	1	1.7
Reports not received until 31.03.2025	7	11.8
Total	59	100

**Table 6:** Distribution of autopsies performed according to duration (from autopsy date) after which viscera report was not received (until 31.03.2025)

Duration (from autopsy date)	Frequency (n=7)	Percentage (%)
> 5 – 6 years	1	14.3
> 6 – 7 years	1	14.3
> 7 years	5	71.4
Total	7	100

Around 76.9% (n=40) of the viscera analysis yielded negative results for the presence of any metallic substances, alkaloids, pesticides, as well as both volatile and non-volatile poisons. Among the viscera reports that were positive for poisons, the predominant finding was ethyl alcohol contaminated with methyl alcohol (13.5% (n=7), followed by organophosphorus pesticides (5.8% (n=3)), and aluminum phosphide/ethyl alcohol (1.9% (n=1) for each) (Table 7).

**Table 7:** Distribution of autopsies performed according to viscera report findings

Viscera report findings	Frequency (n=52)	Percentage (%)
Negative report	40	76.9
Organophosphorus pesticide detected	3	5.8
Aluminium phosphide detected	1	1.9
Ethyl alcohol detected	1	1.9
Ethyl alcohol + methyl alcohol detected	7	13.5
Total	52	100

A significant proportion of the viscera reports, specifically 55% (n=22), in which no metallic, alkaloidal, pesticidal, volatile, or non-volatile toxins were identified, were submitted subsequent to a period of 12 months post-autopsy. Conversely, the majority of the positive viscera reports, accounting for 75% (n=9), which documented the presence of organophosphorus compounds, aluminium phosphide, methyl alcohol, or ethyl alcohol, were reported within the 12-month timeframe following the date of autopsy (Tables 8-9).

**Table 8:** Distribution of viscera reports that were negative for any poison and duration (from autopsy date) after which viscera report was received

Duration (from autopsy date)	Frequency (n=40)	Percentage (%)
Within 6 months	11	27.5
> 6 – 12 months	7	17.5
> 12 - 18 months	3	7.5
> 18 - 24 months	2	5.0
> 24 – 30 months	1	2.5
> 30 – 36 months	3	7.5
> 36 – 42 months	10	25
> 42 – 48 months	2	5.0
> 48 – 54 months	1	2.5
Total	40	100

**Table 9:** Distribution of viscera reports that were positive for any poison and duration (from autopsy date) after which viscera report was received

Duration (from autopsy date)	Frequency (n=12)	Percentage (%)
Within 6 months	2	16.7
> 6 – 12 months	7	58.3
> 12 - 18 months	1	8.3
> 18 - 24 months	2	16.7
Total	12	100

A chi-square test of independence was utilized to analyse the correlation between the period (tracked from the autopsy date) until the viscera report was available from the Forensic Science Laboratory and the results of the viscera report (defined as negative or positive). The analysis revealed a statistically significant relationship between extended delays and negative toxicological findings (χ² = 8.3, df = 3, p = 0.039). The likelihood of obtaining a negative viscera report increases in correlation with the duration after which the viscera report was received since the date of autopsy (Table 10).

**Table 10:** Correlation between duration after which viscera report was received since the date of autopsy and findings of viscera reports (negative or positive for poisons)

Duration (from autopsy date) after which viscera report was received from FSL	Findings of viscera report				Total	
	Negative for poisons		Positive for poisons			
	n	%	n	%	n	%
Within 6 months	11	21.2	2	3.8	13	25
> 6 – 12 months	7	13.5	7	13.5	14	27
> 12 - 18 months	3	5.8	1	1.9	4	7.7
> 24 months	19	36.5	2	3.8	21	40.3
Total	40	77	12	23	52	100

## Discussion

This study provides a systematic appraisal of viscera preservation and toxicological reporting in medical board autopsies, focusing on operational timelines and procedural bottlenecks. Viscera were retained in 28.6% of the 262 autopsies, reflecting selective decision-making by autopsy surgeons and board members when poisoning was suspected. Such discretion is appropriate but highlights the need for clear, evidence-based criteria to guide preservation.

Timely transfer of specimens from the mortuary to the Forensic Science Laboratory (FSL) was a major weakness. Investigating officers had not collected viscera in 21.3% of cases even after five years, and only 10.7% of specimens were collected within one month of autopsy. Extended mortuary storage risks chemical degradation and loss of volatile substances, directly compromising toxicological accuracy [7]. Similar concerns have led some authors to advocate limiting mortuary storage when no foul play is suspected [8]. Documented obstacles include bureaucratic procedures, infrastructure gaps, and poor inter-agency coordination [2,9-12].

Analysis at the FSL was also protracted. Reports were available within six months in only 22% of cases, while 42.5% were delayed beyond one year, and 11.8% remained pending beyond five years. Such delays impede investigation and judicial proceedings, prolonging uncertainty for families and weakening evidentiary strength [12,13].

Negative results predominated (76.9%). Positive detections occurred in 23.1% of cases, most often ethyl alcohol mixed with methyl alcohol (13.5%) and organophosphorus compounds (5.8%). These data suggest that routine preservation of viscera in all suspected cases may not be the most efficient use of resources. More stringent case-selection protocols guided by scene evidence and clinical assessment could reduce unnecessary submissions while maintaining forensic value [1,3,5,11,14-16]. Rapid, on-site screening tests at autopsy may further refine the need for full toxicological work-up [17-19].

A significant association emerged between report delay and negative findings: specimens reported after 12 months were more frequently negative. This may reflect both chemical degradation over time and preferential processing of high-suspicion cases [14,20,21]. The finding underscores the importance of secure long-term storage, robust chain-of-custody tracking, and expedited analytical pipelines [2,10,12,22,23]. Notably, 55% of negative reports were released after 12 months, compared with 75% of positive reports within that period, suggesting differential prioritization alongside systemic inefficiency.

The existing framework appears to inadequately optimize the overall efficacy of forensic practices. Prolonged intervals in both the transmission of preserved viscera and the acquisition of toxicology analyses detract from the practical applicability of forensic toxicology within medicolegal inquiries. Given the restricted percentage of affirmative toxicology results, amending policies concerning viscera preservation to prioritize cases exhibiting heightened suspicion of poisoning or substance abuse may facilitate superior resource distribution and enhance judicial outcomes [1,14,24-27]. Addressing these gaps requires coordinated action: prompt collection and dispatch by investigating agencies, expansion of FSL capacity, and deployment of digital tracking for every stage of specimen handling [28-30]. Establishing regional forensic laboratories within teaching hospitals and strengthening direct linkages between medical colleges and State Forensic Science Laboratories could further reduce turnaround times.

Limitations

Conducted at a government tertiary centre in a state capital housing the State Forensic Science Laboratory, the study reflects a setting not representative of more remote hospitals. Reliance on existing records introduces potential information bias through incomplete documentation and inconsistent timestamps. The absence of standardized quantitative toxicological measurements prevents assessment of analyte concentrations and degradation kinetics. While we demonstrate significant correlation between processing delays and negative results (p = 0.039), the observational design precludes causal inference. Multiple unmeasured confounders, including laboratory workload, personnel changes, and case prioritization protocols, may influence observed associations. Prospective multi-center studies with controlled storage experiments and systematic quantitative analysis are needed to validate these findings and establish causality.

## Conclusions

Viscera preservation for toxicological analysis is vital to justice and public health but is hindered by delays, resource constraints, and systemic inefficiencies. Enhancing its effectiveness requires reducing logistical delays, refining case selection, and ensuring timely analysis. Strengthening inter-agency collaboration, adopting advanced technologies, and optimizing workflows can accelerate toxicological reporting. Policymakers should invest in forensic infrastructure and establish clear prioritization guidelines. Exploring alternative specimens and advanced analytical methods can further improve reliability and efficiency. Addressing these challenges will help maximize the value of viscera preservation in supporting justice.
